# Disclosure of diagnosis by parents and caregivers to children infected with HIV in Hawassa, southern Ethiopia: a multicentre, cross-sectional study

**DOI:** 10.1093/inthealth/ihae044

**Published:** 2024-06-13

**Authors:** Kejela Tari, Merga Dheresa, Lemesa Abdisa, Dawit Abebe, Desalegn Admassu, Sinetibeb Mesfin

**Affiliations:** School of Nursing and Midwifery, College of Health and Medical Science, Dilla University, Dilla, Ethiopia; School of Nursing and Midwifery, College of Health and Medical Science, Haramaya University, Haro Maya, Ethiopia; School of Nursing and Midwifery, College of Health and Medical Science, Haramaya University, Haro Maya, Ethiopia; School of Nursing and Midwifery, College of Health and Medical Science, Jigjiga University, Jigjiga, Ethiopia; Department of Biomedical and Laboratory Sciences, College of Health and Medical Science, Haramaya University, Haro Maya, Ethiopia; School of Nursing and Midwifery, College of Health and Medical Science, Haramaya University, Haro Maya, Ethiopia

**Keywords:** disclosure, HIV, infected children, southern Ethiopia

## Abstract

**Background:**

In recent years, the life expectancy of human immunodeficiency virus (HIV)-infected children has increased with the availability of highly active antiretroviral therapy (ART). Regardless of the clinical recommendations encouraging HIV status disclosure, the practice of caregiver disclosure is frequently challenging due to many constraints associated with caregivers and healthcare personnel. As studies suggest, disclosure of the HIV-positive status of children is low, particularly in sub-Saharan Africa, where the majority of infected children reside. Thus the primary objective of this study was to evaluate the prevalence of HIV-positive status disclosure to infected children and the pertinent factors associated with caregivers of these children. Moreover, unlike previous studies conducted in Ethiopia, this study included children residing in orphanages.

**Methods:**

We assessed HIV-positive status disclosure and associated factors among infected children in Hawassa, southern Ethiopia, from 25 May to 20 July 2021. A facility-based cross-sectional study was conducted in six public health facilities that provide HIV treatment and care. Data were collected from 355 randomly selected caregivers using interviewer-administered questionnaires and record reviews. Binary and multiple logistic regression was used to explore the association between independent variables and the outcome. The adjusted odds ratio (aOR) with 95% confidence interval (CI) was computed to determine the strength of the association and a p-value <0.05 was considered statistically significant.

**Results:**

Of the 355 children, 132 (37.2%) were informed about their HIV-positive status. Being of young age (≤12 y) (aOR 0.52 [95% CI 0.28 to 0.98]), having caregivers who were not familiar with anyone who disclosed children's HIV status (aOR 0.28 [95% CI 0.16 to 0.49]), children with a family that had a primary education (aOR 0.46 [95% CI 0.23 to 0.89]) and being a child who has taken ART for <5 y (aOR 0.47 [95% CI 0.28 to 0.80]) had a significant association with non-disclosure of HIV-positive status to infected children.

**Conclusions:**

The findings show that disclosure of HIV-positive status to infected children is low. This suggests the need to provide support and education to caregivers, facilitate experience-sharing sessions between caregivers who disclosed the HIV status to infected children and implement age-specific disclosure interventions for young children. In addition, it is important to provide support and counselling to the children when their HIV status is disclosed.

## Introduction

Human immunodeficiency virus (HIV) is still a leading cause of mortality and one of the world's most critical public health issues.^1^ As of 2022, of the nearly 39 million people estimated to be living with HIV worldwide and 630 000 deaths, approximately 1.7 million are children <15 y of age, with the majority of infected children residing in sub-Saharan Africa.^2^ Around 613 000 people were living with HIV/acquired immunodeficiency syndrome (AIDS) in Ethiopia in 2020, of which an estimated 42 000 were children <15 y of age.^3^

Disclosure is the term used to describe the situation in which a child is aware of his or her HIV status.^4^ In recent years, the life expectancy of HIV-infected children has increased with the availability of highly active antiretroviral therapy (ART). However, how best to disclose the child's HIV-positive status within and outside of the healthcare system has been a challenge for their parents, caregivers and healthcare practitioners.^5^ Disclosure of HIV status is not a one-time event, but rather a process, involving ongoing discussions about the disease as the child or adolescent matures cognitively, socially, emotionally and sexually.^6^

The World Health Organization (WHO) recommends providing children with age-appropriate information as early as possible and fully disclosing their HIV-positive status by the age of 12.^7^ Failure to disclose HIV status to children can result in delayed access to treatment, non-adherence to treatment and treatment failure.^4^ Disclosing the child's HIV-positive status is important to prevent children from feeling isolated and learning their status accidentally or publicly. There is also evidence that children who are given reasons for needing medication are much more likely to have better viral suppression and treatment adherence and to stay on treatment longer.^5^

Despite clinical recommendations that encourage disclosure of HIV status, the practice of disclosure is often challenging due to many limitations associated with caregivers (parents’ fear of disclosure of family secrets, feelings of guilt) and health workers (lack of training, excessive workload).^4,8^ Studies conducted in Ethiopia showed that the prevalence of HIV status disclosure in Gojjam, Bale and Bahirdar was 33.3%, 28.5% and 31.5%, respectively.^9,10^

The current status of HIV disclosure among children with positive status is not known in Hawassa. Furthermore, unlike other studies conducted in Ethiopia, this study included children living in orphanages. The aim of this study was to determine the prevalence of HIV-positive status disclosure and factors in children undergoing ART treatment in public health facilities in Hawassa.

## Methods

### Study area and period

A facility-based cross-sectional study was conducted from 25 May to 20 July 2021 in six public health facilities in Hawassa, southern Ethiopia. Hawassa is the capital city of the Sidama Region, with a total area of 50 km^2^ and a total population of 315 267, located 273 km south of Addis Ababa (latitude 7°3′N and longitude 38°28′ E; elevation 1708 m [5604 ft] above sea level). It has 2 governmental hospitals and 10 health centres. Of these, 2 hospitals and 4 health centres provide ART services.

### Participants

All caregivers of HIV-positive children ages 6–15 y who receive follow-up care at paediatric ART and care centres at Hawassa health facilities were included. Children who came alone or without a caregiver or parents were excluded for ethical reasons.

All government health institutions in Hawassa providing ART service, i.e. the two hospitals and four health centres, were included. The sample size was selected from the targeted ART centres by using a simple random sampling method. Proportional allocation of the number of cases to participate in the study from each health facility was considered.

### Sample size and sampling procedure

A single population proportion formula was used to calculate the sample size, assuming a 95% confidence level, 5% margin of error, 10% non-response rate and 33.3% proportion of disclosure of HIV status to infected children from a study conducted in east Gojjam, Ethiopia.^9^ Accordingly, the total sample size was 374. All public health facilities in Hawassa that provide ART services were included. The calculated sample size was distributed using proportional allocation to size based on the number of cases. A simple random sampling method was used to select caregivers after obtaining the sampling frame (list of HIV-infected children ages 6–15 y) from the registry book of healthcare facilities providing ART services.

### Data collection tools and procedure

Data were collected using a structured interviewer-administered questionnaire and records review adapted from previous similar studies and appropriately contextualized.^11–13^ The tool includes variables such as sociodemographic characteristics related to caregivers and children, disclosure of HIV-positive status and clinical characteristics of both caregivers and children and HIV-positive status. In addition, the child's medical record was reviewed to determine clinical characteristics such as WHO clinical stage, history of hospitalizations, use of ART, duration of ART, current viral load status, current CD4 count, level of ART compliance and prophylaxis. Six nurses who work in paediatric ART clinics gathered data at the ART centre when caregivers came for their children's follow-up visits.

### Variables

#### Dependent variable

The disclosure was defined when the caregiver, healthcare provider or other person told the child that he/she was infected with HIV.^14^

#### Independent variables

Independent variables included caregiver-related and child-related sociodemographic characteristics and caregiver-related factors such as educational status, family/caregiver type/relationship, family/caregiver HIV status, biological family status (dead or alive) and caregiver/family disclosure status.

#### Data quality control

The questionnaire was first prepared in English and then translated into local languages (Amharic and Sidaamu Afoo) by language experts and back-translated into English to check its consistency. Two days of training on the objectives, content and procedures of data collection were carried out for data collectors. Of the total sample, 5% was pre-tested at Leku Hospital before the actual data collection date and some amendments were made accordingly. Both the principal investigator and the hired supervisors were responsible for providing on-the-spot supportive supervision and conducting daily reviews of the questionnaires.

#### Data processing and analysis

Data were coded, cleaned and entered into a computer using EpiData version 3.1 and exported to SPSS version 20 (IBM, Armonk, NY, USA) for analysis. Frequency and percentage were used to summarize descriptive statistics. The outcome variable disclosure was dichotomized into two categories: disclosed (yes=1) and not disclosed (no=0). A bivariate, crude odds ratio (OR) with 95% confidence interval (CI) was determined to identify the association between each independent variable and the outcome variable using binary logistic regression. The degree of association between independent and dependent variables was assessed using an adjusted odds ratio (aOR) with 95% CI. Finally, variables with a p-value <0.05 in the multivariable logistic regression were considered statistically significant.

## Result

### Sociodemographic characteristics

A total of 355 caregivers of children living with HIV participated in the study with a response rate of 94.9%. A total of 144 (37.7%) of the caregivers were married and 261 (73.5%) were urban residents. The mean age of the caregivers was 39.61±7.45 y and 233 (65.6%) were 30–44 y of age. Among the children living with HIV, 183 (51.5%) were male, and the majority, 229 (64.5%), were below fifth grade. The mean age of the children was 11.4±2.4 y (Table 1).

**Table 1.** Sociodemographic characteristics of caregivers and their perinatally HIV-infected children, east Ethiopia

Characteristics	Category	n (%)
Caregivers		
Sex	Male	158 (44.5)
	Female	197 (55.5)
Relationship with child	Biological parent	208 (58.6)
	Non-biological parent	147 (41.4)
Occupation	Unemployed	15 (4.2)
	Government employee	152 (42.8)
	Merchant	144 (40.6)
	Farmer	31 (8.7)
	Other^a^	13 (3.7)
Religion	Christian	252 (71)
	Muslim	103 (29)
Ethnicity	Sidama	111 (31.3)
	Oromo	86 (24.2)
	Amhara	60 (16.9)
	Wolayta	72 (20.3)
	Other^b^	26 (7.3)
Marital status	Married	134 (37.7)
	Single	41 (11.5)
	Widowed/widower	157 (44.2)
	Divorced	23 (6.5)
Residency	Urban	261 (73.5)
	Rural	94 (26.5)
Children		
Sex	Male	183 (51.5)
	Female	172 (48.5)
Child educational status	Not started education	14 (3.9)
	<5	229 (64.5)
	Grade ≥5	112 (31.5)
Birth order	1	164 (46.2)
	2	142 (40)
	3	31 (8.7)
	4	7 (2.0)
	5	11 (3.1)
Loss of family member to HIV	Yes	146 (41)
	No	209 (59)
Parent/s lost	Mother only	47 (32.2)
	Father only	59 (40.4)
	Both mother and father	40 (27.4)

^a^House wife, daily laborer.

^b^Gurage, Hadiya, Gedeo.

### Clinical characteristics of children and caregivers

In this study, 240 (67.6%) of the caregivers were HIV positive, with 161 (70.6%) having been on ART for >5 y and 131 (54.6%) had disclosed their HIV status to family members or others. Of the 355 reviewed records, 188 (53%) HIV-infected children were diagnosed when they were <5 y of age, and the majority (289 [81.4%]) were in WHO clinical stage I (Table 2).

**Table 2.** Clinical characteristics of children living with HIV, Hawassa health facilities, southern Ethiopia, 2021

Characteristics	Category	n (%)
HIV-positive children		
Current viral load	High	59 (16.6)
	Low	296 (83.4)
	Total	355 (100)
Duration on ART (years)	<5	150 (42.3)
	≥5	205 (57.7)
Current CD4 count	<200	22 (6.2)
	200–500	82 (23.1)
	>500	251 (70.7)
ART adherence	Good	292 (82)
	Fair	34 (10)
	Poor	29 (8)
History of hospitalization	Yes	261 (73.5)
	No	94 (26.5)
Caregivers		
HIV status	Positive	240 (67.6)
	Negative	78 (22)
	Unknown	37 (10.4)
Duration on ART (years)	<5	67 (29.4)
	≥5	161 (70.6)
Caregiver disclosed status	Yes	131 (54.6)
	No	109 (45.4)
If yes, to whom disclosed	Spouse	28 (21.4)
	Children	15 (11.5)
	Relative	78 (59.5)
	Other	10 (7.6)

### Disclosure status of HIV-positive children

Of the 355 children, only 132 (37.2%; 95% CI 32.1 to 42.1) were fully informed of their HIV serostatus and more than half (71 [53.8]) were disclosed by their healthcare provider. A total of 86% of the disclosed children were 12–15 y of age, with the remainder between 9 and 11 y of age. The mean age of the children at the time of disclosure was 11.29±1.21 y. More than half of caregivers (54.1%) said it was important to disclose to their children their HIV status (Table 3).

**Table 3.** HIV status disclosure of children and their caregiver's at Hawassa health facilities, southern Ethiopia

Variables	Category	n (%)
Caregiver perceived importance of disclosure	Yes	192 (54.1)
	No	163 (45.9)
Caregiver perceived reason for not disclosing (n=163)	Child is too young	62 (38)
	Child may not keep secret	28 (17.2)
	Fear of child self-discrimination	43 (26.4)
	Family child relationship may be affected	22 (13.5)
	Child may feel hopeless	8 (4.9)
Caregiver perceived reason for disclosing	Child thought to be matured	114 (59.4)
	Repeated question by the child	19 (9.9)
	Good adhere to medication	38 (19.8)
	Right to know about his/her disease condition	8 (4.2)
	Self-care and prevent unknowingly transmission	7 (3.6)
	To share responsibility and to get relief	6 (3.1)
Caregivers preferred age of disclosure (years)	<10	39 (11)
	10–12	82 (23.2)
	≥ 13	233 (65.8)
Who is responsible for disclosure	Caregiver/parents	177 (50)
	Joint caregiver and health professionals	100 (28)
	Health professionals	78 (22)
Does the child ask you about his/her health condition or why he/she is taking ART drug?	Yes	189 (53.2)
	No	166 (46.8)
If the child asked about taking ART what was your response? (n=189)	I deflect the information	97 (51.3)
	I tell him a lie	55 (29.1)
	I tell him he/she has HIV	30 (15.9)
	Other^a^	7 (3.7)
Did the child miss taking of his/her pills in the last 2 weeks	Yes	73 (20.6)
	No	282 (79.4)
If yes how many doses he/she miss?	1	58 (79.5)
	2	9 (12.3)
	3	6 (8.2)
Reason for missing the pills	Forgetfulness	42 (57.5)
	Child refused to take	13 (17.8)
	Other^b^	18 (24.7)
HIV disclosure to child	Yes	132 (37.2)
	No	223 (62.8)
Age at disclosure (years)	11.35±1.207	
Who made the disclosure (n=132)	Mother	27 (20.5)
	Father	28 (21.2)
	Health care provider	71 (53.8)
	Other family member	6 (4.5)
Was the disclosure before or after starting ART	Before	22 (16.7)
	After	110 (83.3)

^a^I am also taking it, good to kill germ.

^b^When relative (guest) came to their home, move to other place for ceremonies (wedding).

### Factors associated with children HIV status disclosure

In the binary logistic regression analysis, the age of the child, the child's duration on ART, the caregiver's educational status and the caregiver's knowledge of someone who did disclosure were statistically associated with HIV-positive disclosure status. After controlling for confounders, a caregiver was 48% less likely to disclose if the age of the child was ≤12 y (aOR 0.52 [95% CI 0.28 to 0.98]). The likelihood of disclosure of children's HIV status was reduced by about 72% among caregivers who did not know someone who disclosed children's HIV status compared with their counterparts (aOR 0.28 [95% CI 0.16 to 0.49]). Moreover, children with a family who had a primary education were 55% less likely to disclose than those with a family who had a secondary or above education (aOR 0.46 [95% CI 0.23 to 0.89]). Similarly, children who had been on ART drugs for <5 y were 53% less likely to be aware of their HIV status than children who had been on ART for ≥5 y (aOR 0.47 [95% CI 0.28 to 0.80]) (Table 4).

**Table 4.** Factors associated with child HIV status disclosure at Hawassa health facilities, southern Ethiopia, 2021

		Disclosure status (N=355)				
Variables	Categories	No (n=223)	Yes (n=132)	Crude OR (95% CI)	aOR	p-Value
Child age (years)	>12	53	44	1	1	
	≤12	170	88	0.624 (0.388–1.003)	0.524 (0.280–0.980)*	0.043
Residence	Rural	69	25	1.918 (1.140-3.225)	0.655 (0.349-1.230)	0.188
	Urban	154	107	1	1	
Caregiver knows someone who disclosed	Yes	61	73	1	1	
	No	162	59	0.304 (0.194-0.478)	0.277 (0.158-0.485)**	0.000
Presence of social support	No	110	45	1.882 (1.205-2.938)	0.975 (0.529-1.797)	0.936
	Yes	113	87	1	1	
Child birth order	1	87	77	3.983 (0.835-19.002)	2.955 (0.523-16.707)	0.220
	2	97	45	2.088 (0.433-10.059)	1.959 (0.340-11.303)	0.452
	3	26	5	0.865 (0.142-5.270)	1.128 (0.151-8.421)	0.906
	4	4	3	3.375 (0.396-28.745)	7.137 (0.650-78.417)	
		9	2	1	1	
Loss of family member by HIV	No	140	69	1.540 (0.996-2.382)	1.477 (0.836-2.610)	0.179
	Yes	83	63	1	1	
Caregiver educational status	No formal education	54	63	2.419 (1.437-4.071)	1.736 (0.946-3.186)	0.075
	Primary	85	28	0.691 (0.392-1.219)	0.455 (0.232-0.892)* *	0.022
	Secondary or above	84	41	1	1	
Caregiver relationship with child	Biological parent	145	63	1	1	
	Non -biological	78	69	2.036 (1.313-3.157)	1.493 (0.875-2.546)	0.141
Child viral load status	High	179	117	1	1	
	Low	44	15	0.522 (0.278-0980)	0.681 (0.331-1.401)	0.297
Child asked questions about their health status	Yes	101	70	1	1	
	No	122	62	0.733 (0.476-1.129)	0.823 (0.475-1.427)	0.488
Child duration on ART (years)	6–15	148	57	2.596 (1.668–4.042)	2.127 (1.250–3.619)*	0.005
	1–5	75	75	1	1	

Significantat *p<0.05 and **p<0.001.

## Discussion

This study showed that 132 (37.2%) HIV-positive children knew their serostatus. Factors positively associated with disclosure include the age of the child, the child's duration on ART, the caregiver's education status and the caregiver's knowledge of someone who disclosed.

Our findings are in line with a study done in north Gondar (39.5%)^15^ and east Gojjam, Ethiopia (33.3%).^9^ Studies with similar findings were also reported from India (36.7%)^16^ and Malawi (36%).^17^

The magnitude of disclosure in this study was higher compared with studies done in Kenya (26%) and South Africa (27%).^18,19^ This might be attributed to the higher mean age of children. As children become older, they start asking questions regarding their health status and why they take medication. In addition, caregivers often believe that older children are cognitively and emotionally prepared to accept their disease.^20^ However, when compared with studies conducted in India (51.3%) and Uganda (56%), the magnitude of disclosure in this study is low.^8,21^ The disparity might be explained by the fact that two of those studies recruited children 16–18 y of age, for whom disclosure is both encouraged and practical.^8^

In this study, children ≤12 y of age were 48% less likely to be disclosed compared with their counterparts. This finding is consistent with studies conducted in Ethiopia and Uganda.^14,22,23^ This might be because caretakers believe that children >12 y of age are mature enough to understand the disease and hence have a better chance of receiving information about the consequences of having HIV.^18^ Children with a family that did not know someone who disclosed were 72% less likely to disclose than those with caregivers who knew someone who did. This finding is similar to that in studies conducted in Ethiopia and the USA.^14,24^ This is because caregivers’ knowledge of people who disclosed may allow them to share an experience regarding the disclosure process with other caregivers, which may eventually encourage disclosure. Also, knowing someone who is in another person's shoes may be beneficial to alleviate emotional stress following disclosure.^25^

Consistent with a study done in Ethiopia, children with a family with primary education were 55% less likely to disclose than those with a family with secondary education or above.^4^ This is because educated individuals may be better prepared to manage the disclosure process since they are more aware of the benefits of disclosure.^26^

In line with the findings reported by Alemu et al., children who had taken ART for >5 y were positively associated with disclosure.^27,28^ This might be a result of long-term ART use, which encourages children to inquire about their health and the medications they take.^4^

### Limitations

Relying solely on caregivers for disclosure status reporting poses a limitation, as they may have overreported due to social desirability bias. The study was also influenced by recall bias, although efforts were made to minimize it by cross-referencing caregiver-provided information with the child's medical records.

## Conclusions

We found that disclosure of HIV-positive status to infected children is low. This highlights the importance for healthcare workers in HIV/AIDS care and treatment units to guide caregivers on effectively providing age-appropriate information about HIV-positive status to infected children. Additionally, healthcare providers should address any concerns and misconceptions caregivers may have regarding serostatus disclosure. These measures are crucial for ensuring that children living with HIV receive the necessary support and understanding to navigate their health conditions effectively.

## Data Availability

This study includes all necessary data. However, the corresponding author can provide further information upon request.
